# Analyzing the whole-transcriptome profiles of ncRNAs and predicting the competing endogenous RNA networks in cervical cancer cell lines with cisplatin resistance

**DOI:** 10.1186/s12935-021-02239-6

**Published:** 2021-10-12

**Authors:** Huimin Lv, Shanshan Jin, Binbin Zou, Yuxiang Liang, Jun Xie, Suhui Wu

**Affiliations:** 1 Department of Obstetrics and Gynecology, Third Hospital of Shanxi Medical University (Shanxi Bethune Hospital), Shanxi Academy of Medical Sciences, TaiYuan, 030032 China; 2 Department of Pathology & Shanxi Key Laboratory of Carcinogenesis and Translational Research on Esophageal Cancer, Shanxi Medical University, Taiyuan, 030001 China; 3 Shanxi Key Laboratory of Birth Defect and Cell Regeneration, Shanxi Medical University, TaiYuan, 030001 China; 4 Key Laboratory of Cellular Physiology (Shanxi Medical University), Ministry of Education, TaiYuan, 030001 China

**Keywords:** Cervical cancer, Drug resistance, Cisplatin, ceRNA

## Abstract

**Background:**

Cervical cancer (CC) is one of the most common malignant tumors in women. In order to identify the functional roles and the interaction between mRNA and non-coding RNA (ncRNA, including lncRNA, circRNA and miRNA) in CC cisplatin (DDP) resistance, the transcription profile analysis was performed and a RNA regulatory model of CC DDP resistance was proposed.

**Methods:**

In this study, whole-transcriptome sequencing analysis was conducted to study the ncRNA and mRNA profiles of parental SiHa cells and DDP resistant SiHa/DDP cells. Gene ontology (GO) and Kyoto Encyclopedia of Genes and Genomes (KEGG) were performed for pathway analysis based on the selected genes with significant differences in expression. Subsequently, ceRNA network analyses were conducted using the drug resistance-related genes and signal-transduction pathways by Cytoscape software. Furthermore, a ceRNA regulatory pathway, namely lncRNA-AC010198.2/hsa-miR-34b-3p/STC2, was selected by RT-qPCR validation and literature searching. Further validation was done by both dual-luciferase reporter gene assays and RNA pull-down assays. Besides that, the changes in gene expression and biological function were further studied by performing si-AC010198.2 transfection and DDP resistance analyses in the SiHa and SiHa/DDP cells, respectively.

**Results:**

Using bioinformatics and dual-luciferase reporter gene analyses, we found that AC010198.2/miR-34b-3p/STC2 may be a key pathway for DDP resistance in CC cells. Significant differences in both downstream gene expression and the biological function assays including colony formation, migration efficiency and cell apoptosis were identified in AC010198.2 knockdown cells.

**Conclusions:**

Our study will not only provide new markers and potential mechanism models for CC DDP resistance, but also discover novel targets for attenuating it.

**Supplementary Information:**

The online version contains supplementary material available at 10.1186/s12935-021-02239-6.

## Background

Cervical cancer (CC) caused by persistent human papillomavirus (HPV) infection is one of the most common malignant tumors in women worldwide and an important cause of death from cancer [1]. Despite the development and popularity of HPV vaccines, there are still many patients with recurrent, advanced CC [2]. Cisplatin (DDP)-based chemotherapy was included in the standard treatment for patients with advanced CC by the International Federation of Obstetrics and Gynecology (FIGO) [3]. Once the tumor is resistant to DDP, it means that the efficacy of drug is reduced. As a result, the dose of DDP needs to be increased or the chemotherapy needs to be changed, which will bring a series of negative effects. However, DDP resistance is involved in multiple complex mechanisms, including DNA damage repair and activation of tumor cells [4, 5], reduction of apoptosis [6], target mutations or changes [7–9], and overexpression of MDR-related proteins. They can significantly limit the therapeutic effect of CC patients. However, the specific pathogenesis of CC resistance to DDP remains unclear. Thus, further clarification of the mechanism of CC’s resistance to DDP can improve the benefits of treatment for CC patients.

Non-coding RNA (ncRNA) is a type of RNA transcribed through regions of the human genome that does not code protein, including microRNA (miRNA), long non-coding RNA (lncRNA), and circular RNA (circRNA) [10]. At least 75% of the human genome is transcribed into ncRNA [11]. As a small single-stranded ncRNA, miRNA consists of 19 to 25 nucleotides and regulates gene expression mainly by combining with sequence motifs located in the 3′-untranslated region (UTR) of mRNA transcripts [12]. lncRNA is a type of ncRNA with a length of more than 200 nucleotides, which is mainly transcribed by RNA polymerase II. Its characteristics are similar to messenger RNA, with a 5′-cap and a 3′-poly(A) tail [13]. circRNA is a new type of ncRNA with a closed-loop structure by which it can participate in a series of biological and pathological processes in the manner of a sponge that absorbs miRNA, thereby regulating the progression of the disease [14]. In recent years, with the development of bioinformatics methods and high-throughput deep sequencing technology, it has been discovered that more and more ncRNAs play key roles in tumor development, including tumor cell proliferation, migration, metastasis, and DDP resistance. The variable expression levels of ncRNAs are found at different transcription stage in order to regulate the expression of target genes associated with chemoresistance [15]. In the past few years, competitive endogenous RNAs (ceRNAs) have been shown to be a class of potential post-transcriptional regulators that can alter gene expression through miRNA-mediated mechanisms [16]. These ceRNAs, including various types of RNAs, such as mRNAs, lncRNAs, circRNAs, and pseudogenes, have a common miRNA response element (MRE). They can bind miRNAs and regulate the expressions of their target genes in a competing manner, thereby affecting tumor progression and chemoresistance.

It has recently been reported that the emerging ceRNA networks are involved in DDP resistance of CC. For example, the significantly up-regulated miR-499a can promote the progression of CC and DDP resistance by targeting SOX6 [17]. Overexpressed miR-7-5p can increase the DDP resistance of CC cells by targeting BCL2 and PARP-1 to promote autophagy and inhibit cell DNA repair [18]. The highly expressed lncRNA HOXD-AS1 acts as a sponge for miR-130a-3p, up-regulating ZEB1, thereby enhancing the resistance of CC cells to DDP [19]. The up-regulation of lncRNA PCAT6 and miR-543/ZEB1 axis can regulate the DDP resistance of CC cells [20]. The overexpressed lncRNA NCK1-AS1 can reduce DDP-induced apoptosis by targeting the miR-134-5p/MSH2 axis, thereby leading to the DDP resistance of CC [21]. The down-regulation of lncRNA CASC2 and miR-21/PTEN axis may play important roles in the DDP resistance of CC [22]. However, there remains a lack of systematic evidence on the role of ceRNA in CC DDP resistance.

Herein, we used the whole-transcriptome sequencing technology to screen CC cells (SiHa) and their paired DDP resistant cells (SiHa/DDP) to find differentially expressed (DE) lncRNA, circRNA, miRNA, and mRNA. Gene Ontology (GO), and “Kyoto Encyclopedia of Genes and Genomes” (KEGG) approaches were performed to analyze the significant DE mRNAs in CC DDP resistance. Subsequently, through the prediction of online databases, a ceRNA network of lncRNA/circRNA–miRNA–mRNA was constructed. Finally, a ceRNA pathway was selected and verified by RT-qPCR, RNA pull-down, and dual-luciferase reporter gene assays. Here, our findings provide new evidence of potential molecular biomarkers and their co-expression network in CC DDP resistance, revealing some novel targets for the reversal of DDP resistance.

## Methods

### Cell culture

The human CC cell line SiHa was purchased from the National Collection of Authenticated Cell Cultures (Shanghai, China). It was cultured in DMEM (12430054, Gibco, USA) supplemented with 100 U/ml streptomycin, 100 pg/ml penicillin (P1400, Solarbio, China) and 10 % FBS (04-001-1ACS, BI, USA) at 37 °C in cell incubator with 5% CO_2_. Drug resistance cell line was induced in SiHa cells by treating with the DDP (P4394, Sigma, USA) from 0.1 to 8 µM for more than eight months. Finally, SiHa/DDP cell line could grow stably in the DMEM supplemented with 8 µM DDP.

### Cell Counting Kit-8 assay

SiHa and SiHa/DDP cells (1 × 10^4^ cells/well) were seeded into a 96-well plate. After a 24 h incubation, the cells were treated with 100 µl of medium containing different concentrations of DDP. Subsequent to incubation for an additional 24 h we replaced the medium with 100 µl Cell Counting Kit-8 (CK04, Dojindo, Japan) reagent containing 0.5 mg/ml WST-8. After a 2 h incubation, a microplate reader (Molecular Devices, Danaher, USA) was used to measure the absorption value of each well at 450 nm. Each experiment was repeated for three times. The IC50 value was calculated according to a dose-response curve.

### RNA extraction and quality control

The total RNA of two adherent cells was extracted using Trizol reagent (9109, TaKaRa, Japan). The concentration of RNA was detected by Qubit 2.0 fluorometer. After diluting the RNA sample with RNase-free water, it was electrophoresed on a 1% agarose gel at 180 V for 16 min using Agilent Bioanalyzer 2100. The RNA Nano6000 system was used to determine the integrity and purity of the RNA samples.

### Library construction and RNA sequencing

Approximately 3 µg RNA per sample was used as input material for the RNA sample preparations. A small-RNA sequencing library was generated by NEBNext Multiplex Small RNA Library Prep Set for Illumina following manufacturer’s recommendations. Total RNA was used as the starting sample, and the small RNA ends were directly linked to the adapter, followed by reverse transcription synthesis into cDNA. DNA fragments of 140–150 bp were separated by PAGE gel electrophoresis, and the cDNA library was obtained. Finally, library quality was assessed via the Agilent Bioanalyzer 2100 system, and libraries were sequenced on an Illumina NextSeq 500 platform.

For long RNA library construction, ribosomal RNA was firstly removed using an Ribo-off rRNA Depletion Kit (N406, Vazyme, China). Subsequently, the high strand-specificity library was generated by the VAHT Stranded mRNA-seq V2 Library Prep Kit for Illumina (NR601, Vazyme, China). First-strand cDNA was synthesized by random hexamer primers and M-MuLV Reverse Transcriptase (RNaseH-). Second-strand cDNA synthesis was subsequently performed by means of DNA polymerase I and RNase H. Additionally, PCR was performed with Phusion High-Fidelity DNA polymerase, universal PCR primers, and Index (X) Primers. Finally, the PCR products were purified (AMPure XP system), and library quality was assessed on an Agilent Bioanalyzer 2100 system. The Illumina HiSeq NOVA platform was used for the lncRNA, circRNA, and mRNA sequencing.

### Differential expression gene analysis

Trimmomatic (v0.36) was used to get clean reads from raw reads after quality controlling. For miRNA, the reads were mapped to human reference genome (hg19) based on blastn (v2.6.0). The known mature miRNAs were archived from mirBase (v21), and novel miRNAs were discovered by mirDeep2 (v2.0.0.8). miRanda (v3.3a) was used to predict miRNA-mRNA binding for animal. We used an R package edgeR (v3.18.1) to compare the expression levels of miRNA in paired samples. Regarding lncRNA and mRNA, HISAT2 (v2.1.0) was used to align the clean reads using hg19 reference, and it was run with default parameters. The software StringTie (v1.3.3b) was used to calculate Transcripts Per Million (TPM) of both lncRNAs and mRNAs in each sample. And then DEseq (v1.12.4) was used for differential expression analysis of lncRNA and mRNA. In regard to circRNA, the software BWA was used to map to hg19, and the software CIRI2 (v2.06) was utilized to identify circRNAs. Subsequently, the circRNAs with differential expression was analyzed using DESeq2 (v1.26.0) software. In order to obtain the genes with significantly differential expression, the p-value less than 0.05 and fold change larger than 2 or less than 0.5 were considered as filtering cutoff values.

### Analysis of GO and KEGG pathways

GO (http://geneontology.org/) analysis is a functional method that uses clusterProfiler software to enrich the differential expressed genes. It also employs the principle of hypergeometric distribution to find specific biological functions that are significantly related to differential genes [23].

The KEGG database (https://www.kegg.jp/kegg/) integrates genomic, chemical and system function information [24]. The pathway database integrates current molecular interaction networks and contains more advanced gene-function information. We used clusterProfiler software to calculate the enrichment of the differential genes in the KEGG Pathway entry to find the significant enrichment signal pathways.

### Construction of competitive endogenous RNA network

According to the ceRNA hypothesis, if a certain lncRNA can regulate miRNA and the miRNA can regulate one or more targeted mRNAs, the lncRNA may be used as a ceRNA to regulate mRNAs. Therefore, based on the results of high-throughput sequencing data, we predicted whether miRNA bind to mRNA, lncRNA or circRNA by the 3 search platforms miRTarBase (http://www.mirtarbase.mbc.ncm.edu.tw), DIANA-LncBaseV2 (http://www.microma.gr/LncBase) and miRcode (http://www.mircode.org/mircode). And if the correlation between expression of miRNA and mRNA was negative, the mRNA was selected for miRNA-target pair. The same method was used for lncRNA and circRNA. Subsequently, miRNA-mRNA and miRNA-circRNA pairs were used to build circRNA ceRNA network by Cytoscape software (V. 3.7.2). The same method was used for miRNA-lncRNA and miRNA-mRNA pairs. Based on the circRNA and lncRNA expression profiles,we build up-regulated circRNA/lncRNA and down-regulated circRNA/lncRNA ceRNA network separately.

### Real-time quantitative polymerase chain reaction (RT-qPCR)

The total RNA was extracted according to the instruction of Thermo Scientific GeneJET RNA Purification Kit (K0732, Thermo Scientific, USA). Then, cDNA was generated using HiScript III RT SuperMix (R323-01, Vazyme Biotech, China). GAPDH served as the internal reference gene. miRNAs were detected using miRNAs RT-qPCR Kit (NO. B532461, Sangon, China) normalized by U6. All values were calculated by 2^−∆∆Ct^ method. Primer Premier 5.0 software was used to design primers. Three reactions were performed for each gene. The primer sequences were listed in Table 1.

**Table 1:** Primer sequences of the genes tested by RT-qPCR

Gene	Primer
AC010198.2	F: 5′-GCCGCGATTTTTGTGTCCAA-3′ R: 5′-GACACTTCTCGAGGGCGCTT-3′
STC2	F: 5′-GGGTGTGGCGTGTTTGAATG-3′ R: 5′-CTTGAGGTAGCATTCCCGCT-3′
GAPDH	F: 5′-ACCACAGTCCATGCCATCAC-3′ R: 5′-TCCACCACCCTGTTGCTGTA-3′
hsa-miR-34b-3p	F: 5′-AGGCAGTGTAGTTAGCTGATTGC-3′ R: 5′-TGGTGTCGTGGAGTCG-3′
U6	RT5′-CTCAACTGGTGTCGTGGAGTCGGCAATTCAGTTGAGTTTTT TTTTTTTTTAG-3′ F: 5′-GCTTCGGCAGCACATATACTAAAAT-3′ R: 5′-CGCTTCACGAATTTGCGTGTCAT-3′

### Western blot

The CC cells were lysed with RIPA lysis solution. Cell lysates were extracted and the total protein was measured by the BCA (AR0197, BOSTER, China) method, and 20 µg of total protein was separated by 10% SDS-PAGE. We used a constant current of 300 mA to transfer the protein to the PVDF membrane. Nonspecific binding sites on PVDF membranes were sealed with skim milk. Subsequently, the PVDF membrane was incubated with prepared antibody solution overnight at 4 °C (1:500 dilution, ab63057, Abcam). The HRP-conjugated secondary antibody solution (1:5000, HS101-01, TRANS) was incubated for 2 h at room temperature. ImageJ software was used to calculate the band gray value for data analysis. GAPDH (60004, Proteintech, China) was used as the internal reference.

### RNA pull-down and dual-luciferase reporter gene assay

The biotinylated miR-34b-3p-wild type (WT) and miR-34b-3p-mutant (Mut) probes were synthesized by Sangon Biotech (Shanghai, China). The specific process was according to the Magnetic RNA-Protein Pull-Down kit instructions (20,164, Thermo Fisher, USA). The biotinylated has-miR-34b-3p-WT and MUT probes were transfected into SiHa cells which were collected after 48 h and prepared for cell lysate. The streptavidin magnetic beads were pre-washed and incubated with cell lysate under 4 °C overnight. RNA binding complexes were eluted and RT-qPCR was used to analyze the expression of lncAC010198.2 and STC2 mRNA.

The dual-luciferase reporter gene assay was utilized to verify the relationships between AC010198.2 and miR-34b-3p, miR-34b-3p and STC2. Firstly, the pmirGLO-AC010198.2 and pmirGLO-3’UTR cDNA STC2 vector containing the predicted binding region of AC010198.2 and STC2, respectively, were constructed by Sangon Biotech (Shanghai, China). The SiHa cells grown to 80% were trypsinized to prepare cell suspension, and 4 × 10^5^ cells were seeded in 24-well plate. miR-34b-3p mimic or negative control (150 nM) were co-transfected with pmirGLO-AC010198.2-WT/MUT plasmids into the cells using lipofectamine 3000 (L3000150, Invitrogen, USA) for 48 h. The transfection method of pmirGLO-3′UTR cDNA STC2 was similar to pmirGLO-AC010198.2. The dual-luciferase reporter gene detection kit (E1910, Promega, USA) was used to detect the fluorescence value (RLU) with the Renilla luciferase reporter gene as a control.

### Colony formation and Cell migration assays

1 × 10^3^ cells per well were cultured in 6-well plate in a 37 °C, 5% CO_2_ constant-temperature incubator for 7–14 days. They were fixed and stained with 4% polyformaldehyde and crystal violet successively. The cell clump with no less than 50 cells was counted as a single cell colony. The clone formation rate was then calculated. Each experiment was repeated three times.

The cells (1 × 10^5^ cells/well) were seeded into a 6-well plate. After a 6 h incubation, 2.5 µl pipette tip was used to scratch the cellular monolayer. The cell debris was removed by washing with PBS. The cells were fixed and stained with 4% paraformaldehyde and 0.1% crystal violet after 24 and 48 h respectively. The cell status was observed under an inverted microscope, and the cell migration was recorded. ImageJ software was used to plot and compared the relative migration width of cells.

### Cell apoptosis assay

Firstly, the CC cells were digested with trypsin without EDTA. Subsequently, the cell suspension was incubated with binding buffer added 5 µl of Annexin V and 5 µl of PI (KGA108, KeyGEN, China) away from light at room temperature for 15 min. The ratio of apoptosis and necrosis was detected by FACS Calibur flow cytometer according to standard procedure.

### Statistical analysis

SPSS22.0 software and GraghPadPrism7.0 were used to analyze the data of at least three independent experiments. The expression level of each gene was represented as a fold change by 2^−∆∆Ct^ method. Student’s t-test was used to analyze the differences between the two groups, and the significance was defined as p-value < 0.05.

## Results

### Morphological characteristics and drug sensitivity assay in vitro

**Fig. 1:**
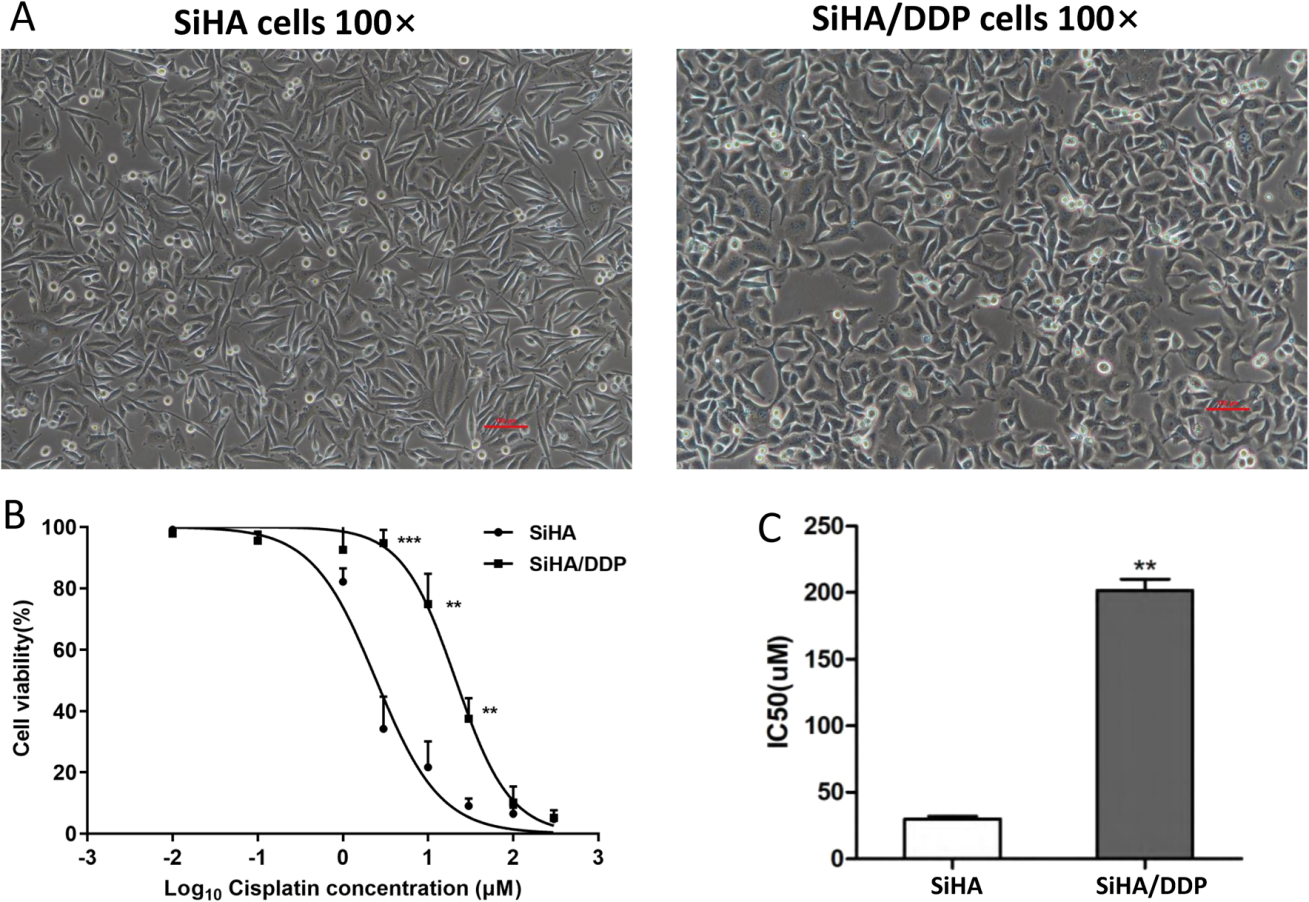
Morphological characteristics and drug sensitivity assay in vitro. **A** The morphological difference between SiHa and SiHa/DDP cells. **B** The cell viability of SiHa and SiHa/DDP cells under different DDP concentrations (0.01 µM, 0.1 µM, 1 µM, 3 µM, 10 µM, 30 µM, 100 µM, 300 µM) were detected using CCK8 assays, and **C** the IC50 of SiHa/DDP cells is significantly higher than that of SiHa cells

SiHa/DDP cells appeared to be polygonal under microscope, while SiHa cells appeared to be spindle-shaped and elongated (Fig. 1A). Thus, it suggested that the increased pseudopodia formation in SiHa/DDP cells may promote the migration and invasion of CC cells.

The resistance was identified by comparing the 50% inhibitory concentration (IC50) value of SiHa/DDP cells with that of SiHa cells. After incubation for 24 h with different concentrations of DDP, the IC50 values ​​of SiHa/DDP and SiHa cells were 212.78 ± 17.89 µM and 27.87 ± 1.72 µM, respectively (Fig. 1B,C). It showed an increase of 7.63 times. The results confirmed that the SiHa/DDP cells were more resistant to DDP than the matched parental SiHa cells.

### Expression profile of circRNAs, lncRNAs, miRNAs and mRNAs

**Fig. 2:**
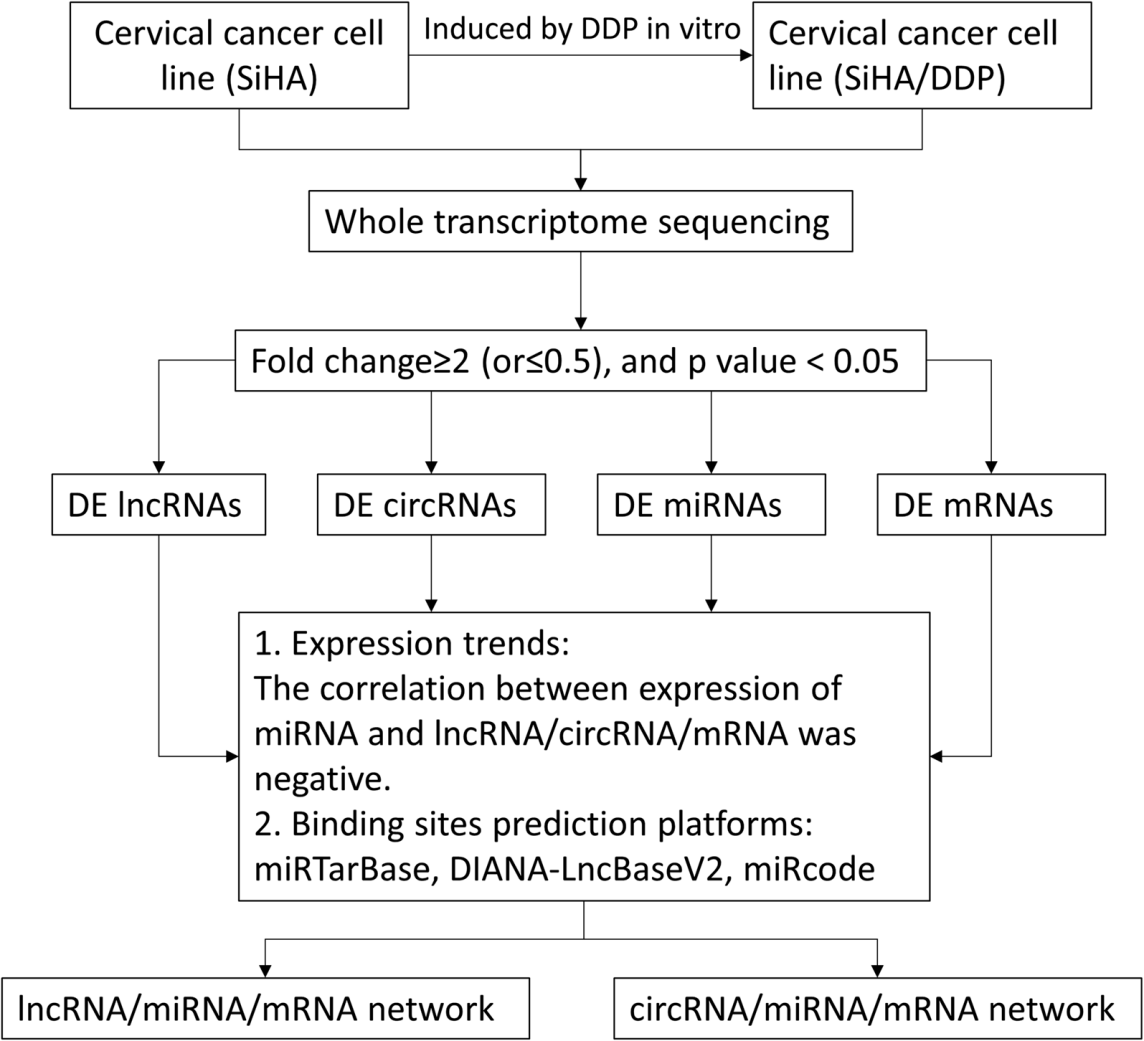
Flow chart of the current study. *DE* differentially expressed

**Fig. 3:**
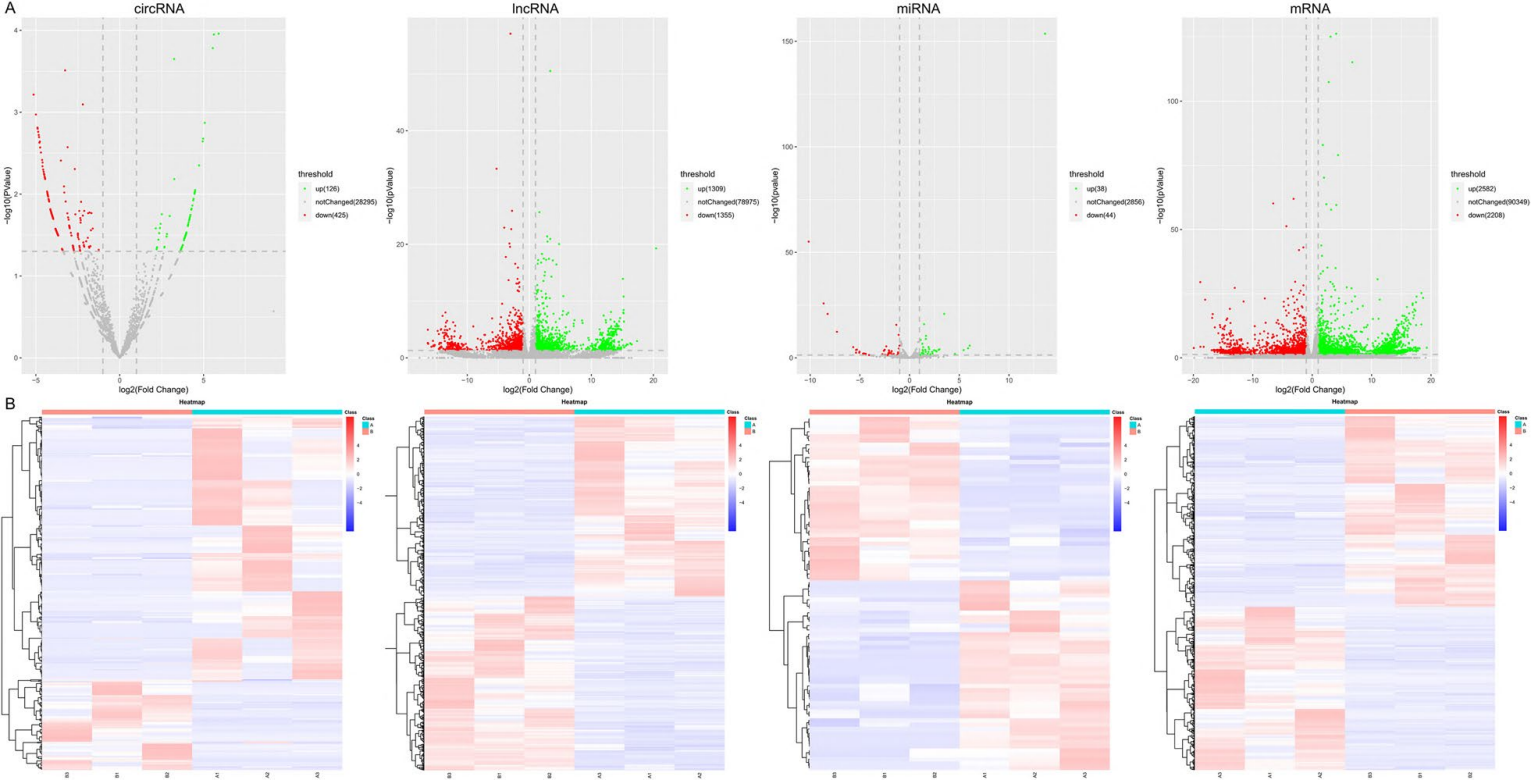
Expression profile of circRNAs, lncRNAs, miRNAs and mRNAs. Volcano map (**A**) and heat map (**B**) show DE circRNAs, lncRNAs, miRNAs and mRNAs in three DDP-sensitive and DDP-resistant CC cell lines. In the volcano map (**A**), the red, green, and gray dots represent the RNAs that are significantly down-regulated, significantly up-regulated, and no significant difference between SiHa/DDP and SiHa cells, respectively. In the heat map (**B**), the red color represents the up-regulated expression, while the blue color represents the down-regulated expression

The whole-transcriptome sequencing data (circRNA, mRNA, lncRNA, miRNA) were obtained from the Illumina Hiseq platform. We analyzed the differential expression (DE) ncRNAs and mRNAs between SiHa/DDP cells and SiHa cells based on the fold change ≥ 2 (or ≤ 0.5) and p value < 0.05. The design and procedure of this current study was displayed in Fig. 2. The volcano map (Fig. 3A) and heat map (Fig. 3B) showed the DE ncRNAs and mRNAs between the two cell lines. The results showed that there were 2664 DE lncRNAs (1309 up-regulated and 1355 down-regulated), 551 DE circRNAs (126 up-regulated and 425 down-regulated), 82 DE miRNAs (38 up-regulated and 44 down-regulated) and 4790 DE mRNAs (2582 up-regulated and 2208 down-regulated).

As shown in Table 2, the top up-regulated lncRNA was lnc-SLC3A2 with a 20.44-fold expression change, and the top down-regulated lncRNA was LINC00852 having a 16.36-fold change. Additionally, the top up-regulated circRNA, miRNA and mRNA were hsa_circRNA_05436 (a 24.91-fold change), hsa-novel-105-mature (a 13.64-fold change) and MORF4L2 (a 19.93-fold change), respectively. The most down-regulated circRNA, miRNA and mRNA were hsa_circRNA_02887 (a 24.83-fold change), hsa-novel-4-mature (a 10.15-fold change) and HLA-A (a 19.28-fold change). Additional file 1: Table S1–S3 listed the top 20 up-regulated and down-regulated lncRNAs, circRNAs and mRNAs. Additional file 1: Table S4 listed the top 10 up-regulated and down-regulated miRNAs.

**Table 2:** The most significant up/down-regulated ncRNAs and mRNAs

DE RNAs	Total no.	No. of upregulated	No. of downregulated	The most upregulated (fold change)	The most downregulated (fold change)
lncRNA	2664	1309	1355	SLC3A2 (20.44)	LINC00852 (16.36)
circRNA	551	126	425	hsa_circRNA_05436 (24.91)	hsa_circRNA_02887 (24.83)
miRNA	82	38	44	hsa-novel-105-mature (13.64)	hsa-novel-4-mature (10.15)
mRNA	4790	2582	2208	MORF4L2 (19.93)	HLA-A (19.28)

### Go and KEGG analysis in DE mRNAs

**Fig. 4:**
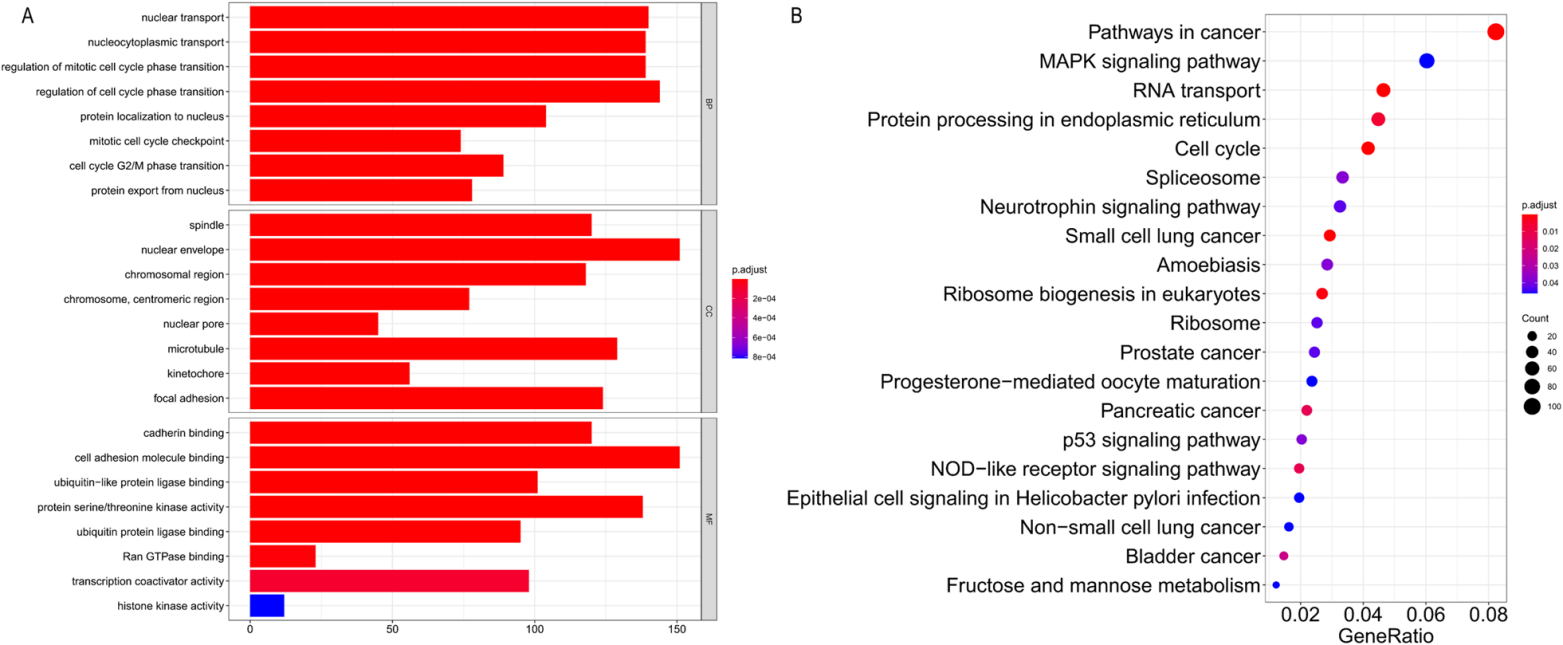
Analysis of GO and KEGG pathways of DE mRNAs

Moreover, we constructed circRNA–miRNA–mRNA network and lncRNA–miRNA–mRNA network. The GO and KEGG pathways were performed for DE mRNA analysis and functional prediction. GO analysis showed that these DE mRNAs of the ceRNA network are mainly related to the spindle (cell component), nuclear transport (biological process) and cadherin binding (molecular function) (Fig. 4A). Enrichment analysis of pathways by KEGG showed that there were 55 pathways in the mRNAs regulated by lncRNA/circRNA–miRNA networks. Of them, pathways in cancer, MAPK signaling pathway and RNA transport were the most abundant pathways (Fig. 4B).

### ceRNA network constructed based on genes related to DDP resistance

As reported before, circRNA or lncRNA can act as a miRNA sponge to manipulate gene expression, thereby playing roles in the development of cancers [25, 26]. The miRTarBase, DIANA-LncBase and miRcode platforms were used to identified the potential RNAs (including mRNAs, lncRNAs and circRNAs), which share the MREs with miRNAs. Then the DE mRNAs related to DDP resistance were selected, and a more concise and effective ceRNA network was discovered.

Based on the sequencing data, we built the lncRNA-miRNA-mRNA and circRNA–miRNA–mRNA interaction networks, respectively. Then, Cytoscape (v3.7.2) was used to for further analysis. The lncRNA–miRNA–mRNA networks included 129 lncRNAs, 37 miRNAs and 333 mRNAs (Additional file 2: Figure S1A). Additionally, the circRNA–miRNA–mRNA networks included 508 circRNAs, 24 miRNAs and 121 mRNAs (Additional file 2: Figure S1B). ceRNA networks have been reported involved in chemoresistance of CC. In order to verify the reliability of the sequencing data, we randomly selected 8 dysregulated lncRNAs from our sequencing data, and performed RT-qPCR methods to validate their expression levels (Additional file 3: Figure S2A, B). We found that lncRNA-AC010198.2 was the most significantly up-regulated, and it can bind 3 DE miRNAs in the ceRNA network. After RT-qPCR verification, we found the 3 miRNAs were significantly down-regulated in SiHa/DDP cells (Additional file 3: Figure S2C). Furthermore, we selected miR-34b-3p as the target of AC010198.2 [27]. In addition, there were 2 DE mRNAs which can bind miR-34b-3p (Additional file 3: Figure S2D). Consequently, we selected STC2 as the target gene of miR-34b-3p according to the previous reports [28–30].

### AC010198.2/hsa-miR-34b-3p/STC2 pathway was verified by RNA pull-down and dual-luciferase reporter gene assays

**Fig. 5:**
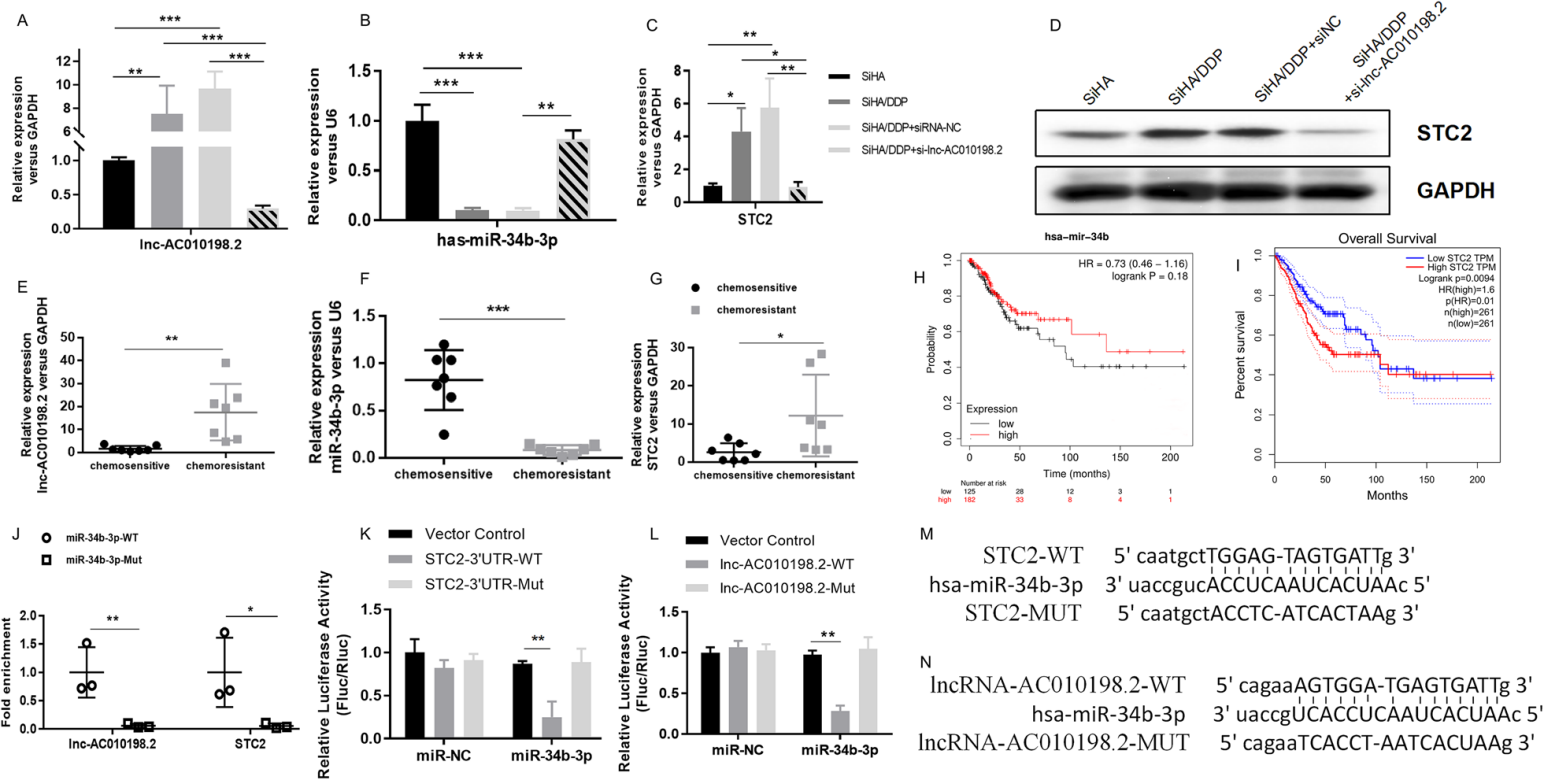
AC010198.2/hsa-miR-34b-3p/STC2 pathway was verified by RNA pull-down and dual-luciferase reporter gene assays. (**A**–**G**) The RNA expression levels of AC010198.2 (**A**, **E**), miR-34b-3p (**B**, **F**) and STC2 (**C**, **G**) in CC cells and tissues (n = 7) were detected by RT-qPCR. **D** The protein level of STC2 in CC cells treated with si-AC010198.2 were detected by Western Blot. **H**–**I**) The highly expressed miR-34b-3p indicated a longer survival time in CESC, and the opposite association with STC2 was observed. **J** RT-qPCR after RNA pull-down assay showed that, in comparison to the miR-34b-3p-Mut-Bio probe, the use of biotin-labeled miR-34b-3p (miR-34b-3p-bio) probe can enrich AC010198.2 and STC2 RNA transcripts. **K** The luciferase activity analysis shows that miR-34b-3p can bind to the 3′UTR of STC2. **L** The luciferase activity analysis shows that miR-34b-3p can bind to AC010198.2. **M** Potential binding sites of STC2' 3'UTR and miR-34b-3p **N** Potential binding sites of miR-34b-3p and AC010198.2

Firstly, the expression levels of AC010198.2, miR-34b-3p and STC2 in chemosensitive and chemoresistant cell lines and tissues were checked. Compared to DDP-resistant CC cells and tissues, the expressions of AC010198.2 and STC2 were significantly increased in chemosensitive cells and tissues (Fig. 5A, C, D, E, G). However, miR-34b-3p showed a significant decrease in expression as indicated (Fig. 5B, F). This was consistent with the RNA sequencing results. Next, we constructed small interfering RNAs (siRNAs) and transfected into the SiHa/DDP cells. Compared with SiHa/DDP-NC, the expression of miR-34b-3p and STC2 showed the highest inhibitory effect after transfection of si-AC010198.2 (Fig. 5A–D). In SiHa/DDP cells, when AC010198.2 was down-regulated, the expression of STC2 mRNA and protein was significantly reduced.

In order to conduct the overall survival analysis of miR-34b-3p and STC2 in cervical squamous cell carcinoma (CESC) patients, we used KAPLAN and GEPIA to perform statistical analysis. We found that the highly expressed miR-34b-3p indicated a longer survival time in CESC (Fig. 5H), and the opposite association with STC2 was observed (Fig. 5I). The bioinformatics prediction tool was further used to prove that miR-34b-3p can target AC010198.2 and 3′UTR of STC2 with complementary binding sites (Fig. 5M, N). The pmirGLO-AC010198.2 and pmirGLO-3’UTR cDNA STC2 vector containing the predicted binding region of AC010198.2 and STC2 were constructed, respectively. Obviously, co-transfection of luciferase reporter plasmid containing WT-AC010198.2 (or WT-STC2) and miR-34b-3p mimics into SiHa cells resulted in a lower value compared with that of miR-NC (Fig. 5K, L). The above results indicated that miR-34b-3p targets AC010198.2 and 3’UTR of STC2 at the predicted MREs. Subsequently, compared with the miR-34b-3p-MUT, the RNA pull-down assay revealed the expression of AC010198.2 and STC2 increased significantly in the miR-34b-3p-WT (Fig. 5J). In summary, the above results confirmed the molecular regulation model of AC010198.2/hsa-miR-34b-3p/STC2 axis and proved the potential regulative role of AC010198.2 in CC DDP resistance.

### The role of AC010198.2 in CC cells

**Fig. 6:**
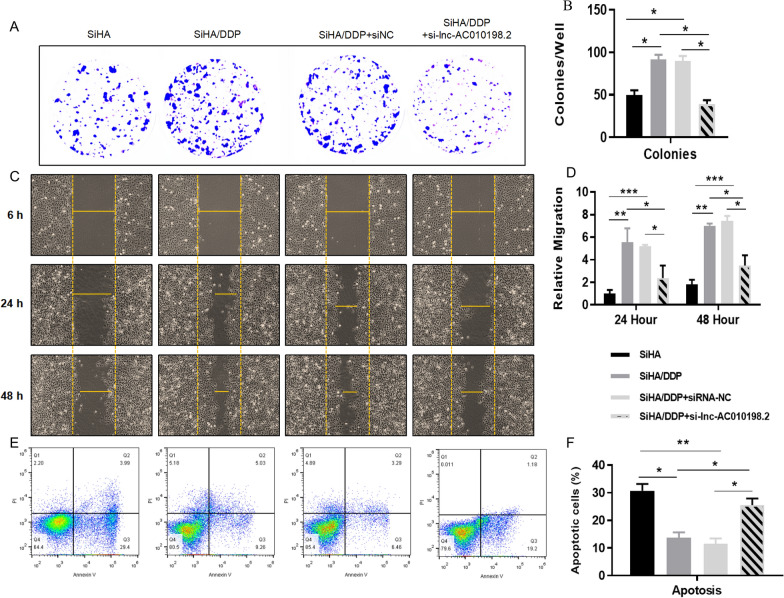
The role of AC010198.2 in CC cells. **A**, **B** The colony formation results are shown to analyse the proliferation of CC cells after transfection with si-AC010198.2. **C**, **D** The cell migration results are shown to compare the migration efficiency of CC cells in different groups. **E**, **F** The apoptotic status of CC cells. The experiments were divided into four groups: SiHa, SiHa/DDP, SiHa/DDP-NC and SiHa/DDP-si-AC010198.2. All data were represented as the mean ± SD. *p < 0.05, **p < 0.01, ***p < 0.001

After si-AC010198.2 was transfected and DDP was treated with IC50 in SiHa and SiHa/DDP cells, respectively, the gene expression and biological function changes were further analyzed to interpret the synergistic effect of AC010198.2 and DDP on tumor cells. Functional assays were performed to test the role of AC010198.2 in colony formation, cell migration and apoptosis of SiHa/DDP cells. Compared with SiHa/DDP and SiHa/DDP-NC cells, SiHa/DDP cells transfected with si-AC010198.2 can significantly inhibit the cell migration and proliferation (Fig. 6A–D). In summary, these findings confirmed that high expression of AC010198.2 promote the proliferation and motility of SiHa/DDP cells, which is a typical feature of cancer stem cells. At the same time, we measured the apoptosis of the four groups of cells by flow cytometry with si-AC010198.2 and DDP treatment as shown in Fig. 6E. Statistical analysis showed apoptosis rate increased in SiHa/DDP cells with si-AC010198.2 (Fig. 6F). In summary, compared with SiHa/DDP cells, DDP significantly promoted the apoptosis of SiHa cells, while the low expression of AC010198.2 can promote the tumor sensitivity to DDP. Its mechanism of action was shown in Additional file 4: Figure S3.

## Discussion

DDP resistance is a main challenge in the treatment of CC [3]. In the past few decades, despite extensive research, the specific mechanism of drug resistance has not been well elucidated yet. More evidences indicate that ncRNAs may play a key role in the resistance of cancer [31–33]. For instance, Huang et al. [34] reported that circAKT3 can act as a sponge of miR-198, promoting the expression of PIK3R1 in gastric cancer and enhancing the tumor resistance to DDP. Zhu et al. [35] found that circPVT1 could promote adriamycin and DDP resistance in osteosarcoma cells by regulating ABCB1. It was reported that circELP3 can promote the proliferation and DDP resistance of bladder cancer cells under hypoxia environment [36]. After screening the DE circRNA profile in pancreatic cancer cells through high-throughput transcriptome sequencing, Shao et al. [37] found two circRNAs which may be related to gemcitabine resistance in pancreatic ductal adenocarcinoma. It was found that circEIF6 can promote the metastasis and DDP resistance of thyroid cancer cells by regulating miR-144-3p/TGF-α axis [38]. Long et al. [39] described that the GAS5-E2F4-PARP1-MAPK axis inhibited the progression and DDP resistance of epithelial ovarian cancer. However, to the best of our knowledge, no systematic research on ncRNA dysregulation in CC chemoresistance has been reported.

In the present study, firstly, we used whole-transcriptome sequencing analysis to compare the expression profiles of ncRNAs between DDP-resistant and sensitive CC cell lines (SiHa/DDP and SiHa). We found that there were 2664 DE lncRNAs (1309 up-regulated and 1355 down-regulated), 551 DE circRNAs (126 up-regulated and 425 down-regulated), 82 DE miRNAs (38 up-regulated and 44 down-regulated) and 4790 DE mRNAs (2582 up-regulated and 2208 down-regulated) based on the fold change ≥ 2 (or ≤ 0.5) and p-value < 0.05. The most dysregulated ncRNAs were circRNA (hsa_circRNA_05436 [+ 24.91], hsa_circRNA_02887 [− 24.83]), lncRNA (SLC3A2 [+ 20.44], LINC00852 [− 16.36]), miRNA (hsa-novel-105 mature [+ 13], hsa-novel-4 mature [− 10]). Subsequently, bioinformatic analyses including GO, KEGG pathways and ceRNA networks analyses were conducted in order to study the biological functions and mechanisms of these ncRNAs in CC chemoresistance. GO enrichment and KEGG pathway analyses showed that DE mRNAs are involved in spindle (cell component), nuclear transport (biological process), cadherin binding (molecular function) and pathways in cancer, MAPK signaling pathway and RNA transport pathway. According to our sequencing results, we found that 2987 lncRNA/miRNA/mRNA regulatory pathways are constructed including 129 lncRNAs, 37 miRNAs and 333 mRNAs, and 4121 cirRNA/miRNA/mRNA/mRNA regulatory pathways are established including 508 circRNAs, 24 miRNAs and 121 mRNAs. In the ceRNA networks, there are 69 up-regulated and 60 down-regulated lncRNAs, 115 up-regulated and 393 down-regulated cirRNAs, 32 up-regulated and 29 down-regulated miRNAs, as well as 229 up-regulated and 224 down-regulated mRNAs. They may be correlated with the regulation of DDP resistance in CC. In addition, the expression changes of lncRNAs from the sequencing data were further validated by RT-qPCR assays. Then lncRNA AC010198.2 was selected for further research due to its high expression in SiHa/DDP cells and chemoresistant tissues.

Actually, there have been several studies confirming that AC010198.2 act as an oncogene to regulate the progression of gastric cancer [40], pancreatic cancer [41], colorectal cancer [42], lung cancer [43], oral squamous cell carcinoma [44] and thyroid cancer [45]. However, the role in cervical cancer or chemoresistance has not been reported yet. In the current study, we found that the expression levels of AC010198.2 and STC2 were significantly upregulated, while miR-34b-3p expression was downregulated in the chemoresistant cells and tissues compared with the chemosensitive groups. After AC010198.2 knockdown and the IC50 treatment of DDP, the biological functions of SiHa and SiHa/DDP cells were further analyzed to assess the synergistic effect of AC010198.2 and DDP on CC cells. The results showed that AC010198.2 could significantly affect cell proliferation, migration and apoptosis of SiHa/DDP cells (Fig. 6), and it has been confirmed that AC010198.2 plays a key role in regulating the DDP resistance of SiHa cells. Dual-luciferase reporter gene and RNA pull-down assays demonstrated that miR-34b-3p could directly bind to AC010198.2 and STC2.

Several studies have proved that circRNAs could regulate miRNA at the transcriptional or post-transcriptional level [46–48]. Chen et al. [49] reported that circRNA_100290 played a role in regulating the miR-29 gene family during the progression of oral cancer. Cheng et al. [50] found that circTP63 affected the progression of lung squamous cell carcinoma by regulating miR-873-3p. It was reported that circHIPK3 can bind to 9 miRNAs in order to regulate target gene expression [51]. Given that miRNAs play a key role in the DDP resistance of CC [52], some lncRNAs may participate in DDP resistance by interacting with miRNAs. Although we identified lnc-AC010198.2 with significantly different expression in this study, more information regarding the circRNA–miRNA–mRNA network is still required for future research. Our study helps the understanding of miRNAs’ upstream and downstream targets as potential CC biomarkers and DDP resistance targets.

## Conclusions

Our study characterized the expression profile of differentially expressed lncRNA/circRNA in DDP-resistant and sensitive CC cell lines. We analyzed the GO and KEGG pathways of DE mRNAs, and constructed ceRNA regulatory networks. The AC010198.2/hsa-miR-34b-3p/STC2 interaction network will help us understand the mechanism of CC DDP resistance and find the novel targets to attenuate it.

## Supplementary Information

**Additional file 1: Table S1.** The top 20 upregulated and downregulated lncRNAs. **Table S2.** The top 20 upregulated and downregulated circRNAs. **Table S3.** The top 20 upregulated and downregulated mRNAs. **Table S4.** The top 10 upregulated and downregulated miRNAs.

**Additional file 2: Figure S1.** ceRNA network constructed based on genesrelated to DDP resistance. The lncRNA-miRNA-mRNA network (A) andcircRNA–miRNA–mRNA network (B) are shown, respectively. The diamonds representlncRNAs, circles represent circRNAs, triangles represent miRNAs and squaresrepresent mRNAs. Up-regulation is indicated by red and down-regulation bygreen.

**Additional file 3: Figure S2.** Verification of the sequencing data. The expression levels of 8 lncRNAs were verifiedin CC cells using RT-qPCR (A). The results showed a strong consistency betweenRT-qPCR and the sequencing data (B). The expression levels of 3 DE miRNAs whichcan potentially bind to AC010198.2 were detected by RT-qPCR in CC cells (C).There were 2 DE mRNAs which can bind miR-34b-3p. Their expression levels wereexamined by RT-qPCR and Western Blot, respectively (D).

**Additional file 4**: **Figure S3.** The action mechanism of AC010198.2 in CC cell lines.

## Data Availability

Availability of data and supporting materials section: Please contact the author for data requests using the E-mail address: doctorwusuhui@163.com
