# Supplementary material for: Analyzing the whole-transcriptome profiles of ncRNAs and predicting the competing endogenous RNA networks in cervical cancer cell lines with cisplatin resistance
Source: Cancer Cell Int. 2021 Oct 12;21:532. doi: 10.1186/s12935-021-02239-6 (PMC8513283; doi:10.1186/s12935-021-02239-6)
Supplement: Supplementary file 1 — Additional file 1: Table S1. The top 20 upregulated and downregulated lncRNAs. Table S2. The top 20 upregulated and downregulated circRNAs. Table S3. The top 20 upregulated and downregulated mRNAs. Table S4. The top 10 upregulated and downregulated miRNAs. [file 12935_2021_2239_MOESM1_ESM.docx]

Table S1

The top 20 upregulated and downregulated lncRNAs

| **lncRNA id-upregulated** | **log2FoldChange** | **pValue** | **qValue** |
| --- | --- | --- | --- |
| ENST00000537508 | 20.43949 | 5.15E-20 | 5.70E-17 |
| ENST00000488391 | 6.03963432 | 2.30E-08 | 4.31E-06 |
| ENST00000482254 | 5.97764337 | 5.24E-05 | 0.003779 |
| ENST00000484515 | 5.34601251 | 2.83E-08 | 5.19E-06 |
| ENST00000535093 | 4.8780769 | 1.32E-07 | 2.03E-05 |
| ENST00000544507 | 4.80940275 | 9.07E-21 | 1.08E-17 |
| ENST00000586280 | 4.50174242 | 5.43E-05 | 0.003894 |
| ENST00000639159 | 4.37851162 | 3.53E-17 | 2.67E-14 |
| ENST00000418546 | 4.28986689 | 1.37E-05 | 0.001222 |
| ENST00000494317 | 3.90003489 | 1.71E-08 | 3.30E-06 |
| ENST00000550515 | 3.78803578 | 6.85E-18 | 5.77E-15 |
| ENST00000483500 | 3.56579304 | 4.91E-15 | 2.80E-12 |
| ENST00000559776 | 3.44637033 | 4.81E-05 | 0.003523 |
| ENST00000498644 | 3.37571118 | 1.13E-21 | 1.55E-18 |
| ENST00000503234 | 3.37130489 | 2.98E-51 | 1.96E-47 |
| ENST00000547169 | 3.29481086 | 3.17E-11 | 1.02E-08 |
| ENST00000490885 | 3.16566621 | 3.64E-18 | 3.18E-15 |
| ENST00000575001 | 2.94094116 | 3.34E-21 | 4.27E-18 |
| ENST00000551972 | 2.87672863 | 4.10E-22 | 6.03E-19 |
| ENST00000486054 | 2.71862126 | 4.35E-07 | 5.97E-05 |
| **lncRNA id-downregulated** | **log2FoldChange** | **pValue** | **qValue** |
| ENST00000475197 | -16.358674 | 1.12E-05 | 0.001019 |
| ENST00000480865 | -5.9949819 | 1.12E-07 | 1.77E-05 |
| ENST00000427111 | -5.2755544 | 4.88E-34 | 2.09E-30 |
| ENST00000528834 | -4.0398927 | 1.18E-23 | 2.16E-20 |
| ENST00000562946 | -3.7879743 | 1.69E-18 | 1.56E-15 |
| ENST00000569377 | -3.7333306 | 0.00011 | 0.006908 |
| ENST00000515795 | -3.3379434 | 2.06E-14 | 1.12E-11 |
| ENST00000560639 | -3.2285552 | 3.73E-08 | 6.65E-06 |
| ENST00000461356 | -3.1422163 | 3.54E-06 | 0.000382 |
| ENST00000556324 | -3.1023852 | 2.80E-20 | 3.17E-17 |
| ENST00000460124 | -3.0763357 | 9.35E-09 | 1.93E-06 |
| ENST00000471872 | -3.0398065 | 8.05E-58 | 6.11E-54 |
| ENST00000504789 | -3.0368635 | 1.08E-08 | 2.18E-06 |
| ENST00000605322 | -2.946764 | 9.23E-06 | 0.000868 |
| ENST00000550557 | -2.9063606 | 2.11E-23 | 3.63E-20 |
| ENST00000609207 | -2.7801532 | 1.25E-26 | 3.08E-23 |
| ENST00000463312 | -2.3689734 | 7.38E-05 | 0.004985 |
| ENST00000521778 | -2.2848907 | 3.77E-06 | 0.000407 |
| ENST00000468280 | -2.2623867 | 2.77E-17 | 2.15E-14 |
| ENST00000470036 | -2.2621875 | 0.000173 | 0.00998 |

Table S2

The top 20 upregulated and downregulated circRNAs

| **circRNA id-upregulated** | **log2FoldChange** | **pValue** | **qValue** |
| --- | --- | --- | --- |
| circRNA05436 | 24.91351 | 0.180555 | 0.866015 |
| circRNA04307 | 24.62821 | 0.040519 | 0.866015 |
| circRNA04338 | 24.09498 | 0.004917 | 0.866015 |
| circRNA05445 | 23.87456 | 0.31268 | 0.866015 |
| circRNA04505 | 23.82731 | 0.008353 | 0.866015 |
| circRNA04108 | 23.80623 | 0.016314 | 0.866015 |
| circRNA04522 | 23.72021 | 0.485967 | 0.866015 |
| circRNA06050 | 23.71563 | 0.278888 | 0.866015 |
| circRNA04401 | 23.69901 | 0.304037 | 0.866015 |
| circRNA04331 | 23.40584 | 0.117041 | 0.866015 |
| circRNA03908 | 23.3319 | 0.328811 | 0.866015 |
| circRNA03846 | 23.29103 | 0.41403 | 0.866015 |
| circRNA05078 | 23.28447 | 0.5054 | 0.866015 |
| circRNA05624 | 23.26738 | 0.450042 | 0.866015 |
| circRNA03867 | 23.25001 | 0.286022 | 0.866015 |
| circRNA03918 | 23.236 | 0.269159 | 0.866015 |
| circRNA05391 | 23.23103 | 0.270147 | 0.866015 |
| circRNA04218 | 23.1872 | 0.222376 | 0.866015 |
| circRNA04252 | 23.17861 | 0.158369 | 0.866015 |
| circRNA03889 | 23.11196 | 0.01244 | 0.866015 |
| **circRNA id-downregulated** | **log2FoldChange** | **pValue** | **qValue** |
| circRNA02887 | -24.8335 | 0.186191 | 0.866015 |
| circRNA00333 | -24.0538 | 0.230998 | 0.866015 |
| circRNA01984 | -23.909 | 0.205305 | 0.866015 |
| circRNA02578 | -23.7906 | 0.165156 | 0.866015 |
| circRNA00552 | -23.7334 | 0.141428 | 0.866015 |
| circRNA02044 | -23.7307 | 0.212222 | 0.866015 |
| circRNA03036 | -23.6835 | 0.083926 | 0.866015 |
| circRNA01166 | -23.5532 | 0.240705 | 0.866015 |
| circRNA01478 | -23.5409 | 0.003219 | 0.866015 |
| circRNA02108 | -23.4718 | 0.262347 | 0.866015 |
| circRNA01254 | -23.442 | 0.222769 | 0.866015 |
| circRNA00318 | -23.4218 | 0.268819 | 0.866015 |
| circRNA02119 | -23.3744 | 0.374988 | 0.866015 |
| circRNA00243 | -23.3326 | 0.306993 | 0.866015 |
| circRNA02885 | -23.3082 | 0.422028 | 0.866015 |
| circRNA02641 | -23.2793 | 0.245538 | 0.866015 |
| circRNA01983 | -23.2433 | 0.446264 | 0.866015 |
| circRNA03811 | -23.2389 | 0.45277 | 0.866015 |
| circRNA03032 | -23.2191 | 0.45277 | 0.866015 |
| circRNA02782 | -23.199 | 0.232324 | 0.866015 |

Table S3

The top 20 upregulated and downregulated mRNAs

| **mRNA id- upregulated** | **log2FoldChange** | **pValue** | **qValue** |
| --- | --- | --- | --- |
| ENST00000396634 | 19.27784 | 0.000118 | 0.0073 |
| ENST00000225474 | 18.6552 | 4.68E-24 | 9.23E-21 |
| ENST00000393888 | 18.37913 | 5.71E-26 | 1.34E-22 |
| ENST00000424880 | 18.36353 | 9.89E-14 | 4.73E-11 |
| ENST00000557843 | 17.98278 | 0.000153 | 0.009072 |
| ENST00000326799 | 17.73859 | 0.000168 | 0.009702 |
| ENST00000542877 | 17.55325 | 1.32E-24 | 2.71E-21 |
| ENST00000323871 | 17.48673 | 1.72E-13 | 7.91E-11 |
| ENST00000424225 | 17.46828 | 2.27E-22 | 3.49E-19 |
| ENST00000533909 | 17.38785 | 6.80E-05 | 0.004675 |
| ENST00000243213 | 17.35006 | 4.57E-19 | 4.42E-16 |
| ENST00000360319 | 17.3443 | 0.000103 | 0.006544 |
| ENST00000299502 | 17.18205 | 1.27E-19 | 1.31E-16 |
| ENST00000529029 | 17.15693 | 2.45E-17 | 1.94E-14 |
| ENST00000628067 | 17.10055 | 0.000121 | 0.007447 |
| ENST00000572789 | 17.02287 | 4.00E-16 | 2.64E-13 |
| ENST00000508549 | 16.93388 | 1.64E-18 | 1.53E-15 |
| ENST00000230510 | 16.91181 | 6.66E-22 | 9.36E-19 |
| ENST00000558964 | 16.90595 | 2.67E-21 | 3.51E-18 |
| ENST00000538265 | 16.85939 | 1.10E-07 | 1.74E-05 |
| **mRNA id-downregulated** | **log2FoldChange** | **pValue** | **qValue** |
| ENST00000423833 | -19.9274 | 0.000146 | 0.008686 |
| ENST00000502949 | -18.8331 | 3.03E-30 | 1.03E-26 |
| ENST00000526390 | -18.8198 | 4.92E-05 | 0.003592 |
| ENST00000620121 | -18.2566 | 5.19E-05 | 0.003755 |
| ENST00000617412 | -18.014 | 2.20E-23 | 3.67E-20 |
| ENST00000453664 | -16.8316 | 2.78E-16 | 1.89E-13 |
| ENST00000449224 | -16.7419 | 9.20E-18 | 7.49E-15 |
| ENST00000389690 | -16.3887 | 1.40E-12 | 5.68E-10 |
| ENST00000380393 | -15.7928 | 2.49E-10 | 7.01E-08 |
| ENST00000451903 | -13.0399 | 5.56E-28 | 1.61E-24 |
| ENST00000620225 | -11.545 | 1.15E-22 | 1.86E-19 |
| ENST00000618395 | -9.66914 | 4.61E-13 | 1.98E-10 |
| ENST00000329331 | -9.63118 | 5.11E-08 | 8.79E-06 |
| ENST00000310483 | -8.9571 | 1.74E-07 | 2.62E-05 |
| ENST00000334478 | -8.91479 | 1.22E-05 | 0.0011 |
| ENST00000502553 | -8.78676 | 0.000121 | 0.007454 |
| ENST00000570337 | -8.76984 | 4.13E-10 | 1.11E-07 |
| ENST00000359337 | -8.3634 | 1.12E-07 | 1.77E-05 |
| ENST00000618940 | -7.97384 | 7.23E-24 | 1.37E-20 |
| ENST00000629042 | -7.65553 | 3.29E-06 | 0.00036 |

Table S4

The top 10 upregulated and downregulated miRNAs

| **Id-upregulated** | **log2FoldChange** | **log2CPM** | **pvalue** |
| --- | --- | --- | --- |
| hsa-novel-105-mature | 13.63586233 | 8.366113 | 2.59E-154 |
| hsa-novel-124-mature | 6.042755805 | 1.177667 | 1.60E-06 |
| hsa-novel-165-mature | 5.883068465 | 1.081149 | 2.87E-05 |
| hsa-novel-51-mature | 5.43663243 | 0.814388 | 0.00021381 |
| hsa-novel-153-mature | 4.576690441 | 0.411377 | 0.02980639 |
| hsa-novel-116-mature | 4.567909752 | 0.410906 | 0.013766 |
| hsa-novel-145-mature | 3.483299059 | 3.835335 | 1.46E-21 |
| hsa-miR-3960 | 3.014472207 | 1.260917 | 9.10E-05 |
| hsa-miR-20b-3p | 2.929497844 | 1.217763 | 0.00016227 |
| hsa-novel-93-mature | 2.719137262 | 1.344837 | 0.00084316 |
| **Id-downregulated** | **log2FoldChange** | **log2CPM** | **pvalue** |
| hsa-novel-4-mature | -10.15004376 | 5.010817 | 8.67E-56 |
| hsa-novel-162-mature | -8.642268546 | 3.620544 | 1.64E-26 |
| hsa-novel-157-mature | -8.239079741 | 3.263474 | 1.56E-21 |
| hsa-novel-161-mature | -7.305716774 | 2.460059 | 4.49E-13 |
| hsa-novel-126-mature | -5.667120939 | 1.225224 | 8.50E-06 |
| hsa-miR-145-5p | -5.383734385 | 1.006888 | 0.00364359 |
| hsa-novel-125-mature | -5.378461442 | 1.04585 | 0.00018125 |
| hsa-novel-175-mature | -5.299169249 | 0.997594 | 0.00011541 |
| hsa-novel-58-mature | -5.057457654 | 0.846413 | 0.00375344 |
| hsa-novel-67-mature | -5.037102442 | 0.844854 | 0.00125788 |
