# Supplementary figures and images for: Analyzing the whole-transcriptome profiles of ncRNAs and predicting the competing endogenous RNA networks in cervical cancer cell lines with cisplatin resistance
Source: Cancer Cell Int. 2021 Oct 12;21:532. doi: 10.1186/s12935-021-02239-6 (PMC8513283; doi:10.1186/s12935-021-02239-6)

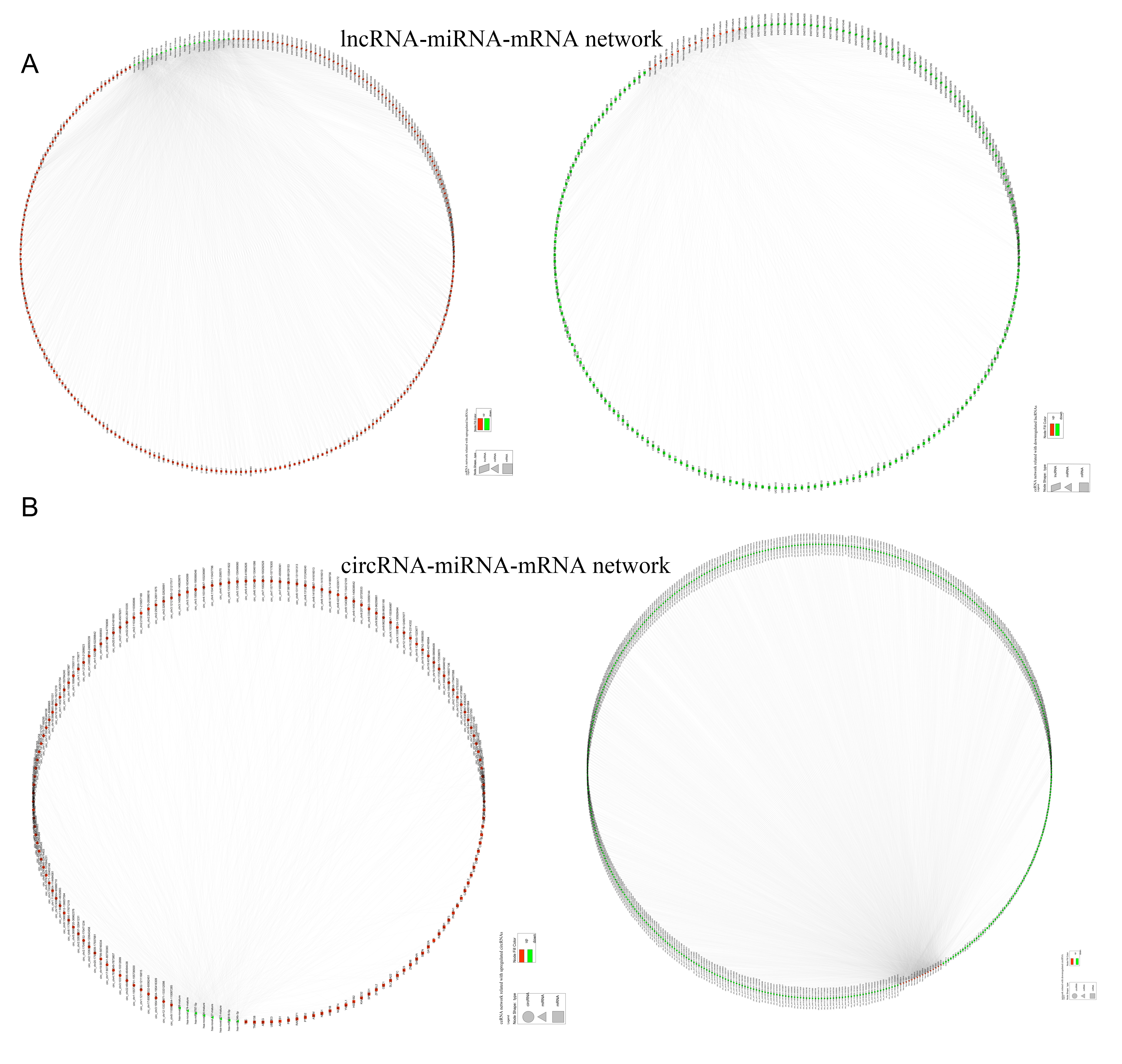

Supplement: Supplementary file 2 — Additional file 2: Figure S1. ceRNA network constructed based on genesrelated to DDP resistance. The lncRNA-miRNA-mRNA network (A) andcircRNA–miRNA–mRNA network (B) are shown, respectively. The diamonds representlncRNAs, circles represent circRNAs, triangles represent miRNAs and squaresrepresent mRNAs. Up-regulation is indicated by red and down-regulation bygreen. [file 12935_2021_2239_MOESM2_ESM.tif]

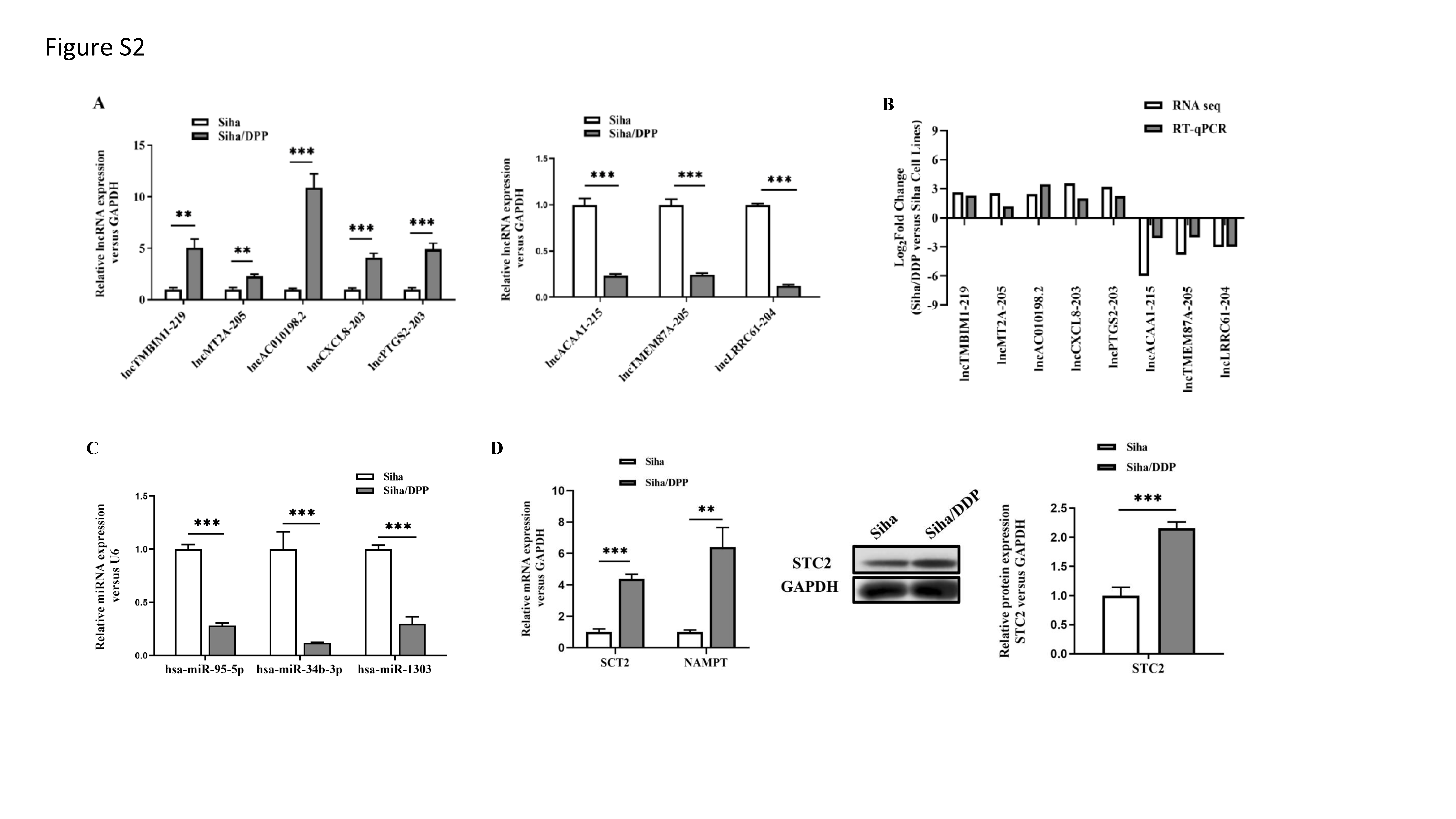

Supplement: Supplementary file 3 — Additional file 3: Figure S2. Verification of the sequencing data. The expression levels of 8 lncRNAs were verifiedin CC cells using RT-qPCR (A). The results showed a strong consistency betweenRT-qPCR and the sequencing data (B). The expression levels of 3 DE miRNAs whichcan potentially bind to AC010198.2 were detected by RT-qPCR in CC cells (C).There were 2 DE mRNAs which can bind miR-34b-3p. Their expression levels wereexamined by RT-qPCR and Western Blot, respectively (D). [file 12935_2021_2239_MOESM3_ESM.tif]

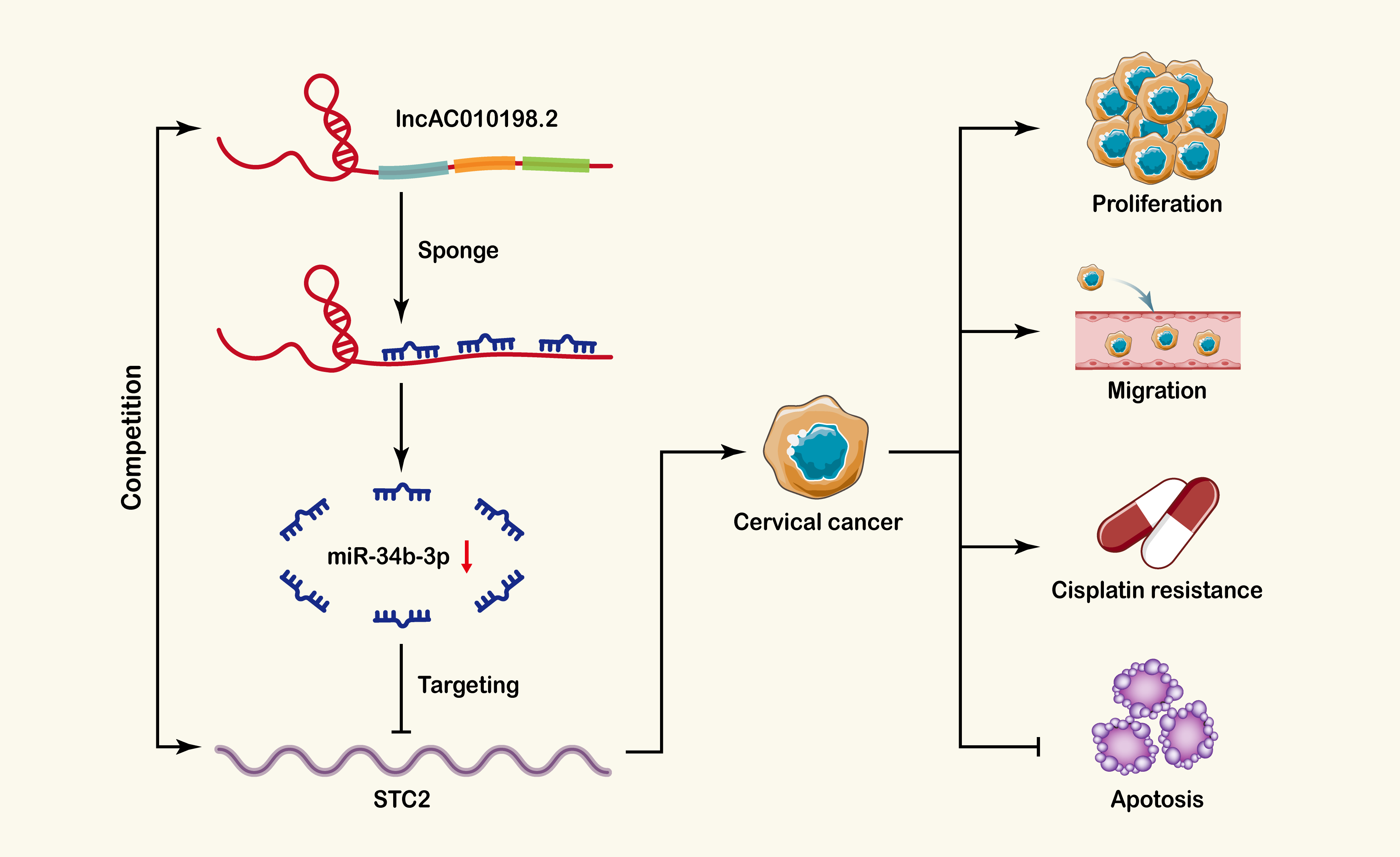

Supplement: Supplementary file 4 — Additional file 4: Figure S3. The action mechanism of AC010198.2 in CC cell lines. [file 12935_2021_2239_MOESM4_ESM.tif]
